# Retrospective Screening for Zoonotic Viruses in Encephalitis Cases in Austria, 2019–2023, Reveals Infection with Lymphocytic Choriomeningitis Virus but Not with Rustrela Virus or Tahyna Virus

**DOI:** 10.3390/v17030300

**Published:** 2025-02-21

**Authors:** Jeremy V. Camp, Norbert Nowotny, Stephan W. Aberle, Monika Redlberger-Fritz

**Affiliations:** 1 Center for Virology, Medical University of Vienna, 1095 Vienna, Austria; stephan.aberle@meduniwien.ac.at (S.W.A.); monika.redlberger@meduniwien.ac.at (M.R.-F.); 2 Department of Biological Sciences and Pathobiology, Center of Pathobiology, University of Veterinary Medicine Vienna, 1210 Vienna, Austria; norbert.nowotny@vetmeduni.ac.at; 3 Department of Basic Medical Sciences, College of Medicine, Mohammed Bin Rashid University of Medicine and Health Sciences, Dubai Health, Dubai P.O. Box 505055, United Arab Emirates

**Keywords:** lymphocytic choriomeningitis virus, Tahyna virus, rustrela virus, zoonotic virus, encephalitis

## Abstract

Zoonotic viruses may be neglected as etiologies of meningoencephalitis in humans. We performed retrospective testing of cerebrospinal fluid from encephalitis cases in biobank material for three zoonotic or potentially zoonotic viruses: rustrela virus (*Rubivirus strelense*, *Matonaviridae*); Tahyna virus (*Orthobunyavirus tahynaense*, *Peribunyaviridae*); and lymphocytic choriomeningitis virus (“LCMV”, *Mammarenavirus choriomeningitidis*, *Arenaviridae*). The cohort consisted of 443 samples, received for routine diagnostic testing year-round between January 2019 and February 2023, and were negative for herpes simplex viruses, varicella zoster virus, and enteroviruses. Using published RT-qPCR protocols, we did not detect rustrela virus or Tahyna virus in any sample. Using a herein described RT-qPCR protocol, we detected LCMV in one sample. Partial genetic sequencing of the virus suggested that the virus was locally acquired. Our study provides information about the incidence of these viruses in humans in Austria when encephalitis is suspected.

## 1. Introduction

Zoonotic disease monitoring and surveillance is important to better understand the risks posed to public health [1]. A large cohort involving 68 centers in seven European countries (including two in Austria) investigating outcomes of patients with severe meningoencephalitis over 3 years found that 23.7% of cases were due to infectious origin, of which 17.1% were viral, and 26.2% of unknown origin [2]. Similarly, a recent retrospective study of encephalitis cases across three tertiary care hospitals over 10 years in Austria reported 10% with unknown and 73% with infectious etiologies [3]. Moreover, in that study, 21 of the 65 patients classified as having a viral etiology were listed as “undetermined” and 13 of the 108 classified as “infectious etiology” were listed as other/undetermined infectious pathogen, suggesting an additional 30% (34/108) of encephalitis cases were of unknown infectious etiology [3].

The principal viral infections clinicians should consider in cases of encephalitis are typically herpes simplex virus 1, varicella zoster virus, enteroviruses, and human immunodeficiency virus [4]. Although vector-borne and zoonotic agents are underrepresented in diagnostic testing, one study has shown that they were more frequently identified compared to viruses more commonly considered to be associated with encephalitis [5]. This suggests that there is a lack of awareness of zoonotic viruses as potential etiologies of encephalitis. In Austria, several vector-borne and zoonotic viruses should be considered secondarily or depending on epidemiological context (e.g., time of year) in cases of encephalitis: the vector-borne arboviruses (Toscana phlebovirus, West Nile or Usutu flaviviruses, tick-borne encephalitis virus) or mammal-/insectivore-borne viruses (borna disease virus) [4,6,7].

The incidence of some zoonotic viruses known to be endemic in Austria is currently unknown. For example, rustrela virus (“RusV”, *Rubivirus strelense*) is a recently characterized positive-sense RNA virus, closely related to rubella virus in the family *Matonaviridae*. RusV has been associated with the long-recognized “staggering disease” in cats [8], and subsequently has been associated with neuroinvasive disease and encephalitis in many mammals [9,10]. The virus is endemic in Austria [11], and is likely maintained in native rodent hosts [12]. No human cases of RusV have ever been reported.

In contrast to RusV, the rodent-borne lymphocytic choriomeningitis virus (“LCMV”, *Mammarenavirus choriomeningitidis*, family *Arenaviridae*) is a segmented ambisense RNA virus that has been associated with neurovirulence in humans. First isolated from a house mouse (*Mus musculus*) in the USA, 1935, LCMV has historically caused outbreaks of meningitis and encephalitis in the USA and Europe [13,14]. With the exception of congenital and transplantation-related cases [15,16], few cases of LCMV have been reported worldwide since then, despite the demonstration that the virus is actively circulating in wild rodents worldwide [17,18,19]. LCMV has been recently detected in rodents in Austria [20] and nearby on the border between Germany and Czechia [21]. Notably, a laboratory strain of LCMV was detected in Japan from a wild-derived mouse strain originating from Austria [22]. Seroconversion has been detected in zoo workers in Vienna, Austria [23], forest workers and hunters in Italy [18,24], and patients in Hungary [25,26]. However, to our knowledge, no acute human cases of LCMV infection have ever been reported from Austria.

Tahyna virus (“TAHV”, *Orthobunyavirus tahynaense*, family *Peribunyaviridae*) is a minus-sense segmented RNA virus that is transmitted by mosquitoes [27]. It has a relatively wide geographic distribution, and is known to be present in mosquitoes in central and southern Europe [28,29,30,31] as well as China [32,33], and seroconversion of wildlife in Europe suggests that the virus has a complex ecological maintenance potentially involving multiple vertebrate hosts [34,35]. Human cases were originally detected in Czechia, where the disease was associated with mild flu-like symptoms to more severe meningitis or meningoencephalitis [36,37,38]. Seroconversion in humans has more recently been demonstrated in Czechia, Austria, and Croatia [39,40,41], although the precise association with disease remains unclear. Experimental infections have shown that TAHV is neurovirulent and neuroinvasive in rhesus macaques and laboratory mice, depending on strain [42]. Closely related viruses in the California encephalitis serogroup are known to cause meningitis or meningoencephalitis [43,44]. Nonetheless, to our knowledge, no human cases of TAHV have ever been reported from Austria in the 50 years since it was first identified.

To establish the incidence of these zoonotic or potentially zoonotic viruses in Austria, specifically in cases where encephalitis was suspected, we retrospectively screened biobanked cerebrospinal fluid (CSF) samples using molecular methods.

## 2. Materials and Methods

### 2.1. Sample Cohort

To assemble the cohort, we drew upon our biobank of samples, accumulated over time as the national reference laboratory for arboviruses and other viruses, as well as a routine clinical diagnostic laboratory for suspected viral infections serving Vienna and eastern Austria. We queried the biobank for cerebrospinal fluid (CSF) samples from January 2019 to February 2023 where clinical symptoms indicated possible meningitis, meningoencephalitis, or encephalitis (as indicated by the attending physician). In addition to including samples where the physician specifically indicated “encephalitis”, we included samples from patients where symptoms included gait abnormality, limb paralysis, neuropathy, status epilepticus, seizure, aphasia, ataxia, dementia, dysesthesia, or vigilance disorder, sometimes in addition to more nonspecific symptoms (e.g., fever, headache, vomiting, somnolence). We also included samples where the physician specifically requested testing for tick-borne encephalitis virus. Typically, these cases are tested for our standard panel of neurotropic viruses, including Herpes simplex virus-1 (*Simplexvirus humanalpha1*), Herpes simplex virus-2 (*Simplexvirus humanalpha2*), Varicella zoster virus (*Varicellavirus humanalpha3*), or enteroviruses (*Picornaviridae*). Endemic arbovirus infections from tick-borne encephalitis virus, West Nile virus, or Usutu virus were also tested during summer and early fall. Collection date and city of residence were recorded for each sample; otherwise, no additional identifying information was considered. Apart from the establishment of the biobank (approved by the ethics commission of the Medical University of Vienna, EK1513/2016 and EK1035/2016), no ethical approval was required.

### 2.2. Nucleic Acid Extraction and Amplification

Total nucleic acids were extracted from CSF using automated easyMag^®^ NucliSENS^®^ kits (bioMérieux Austria, Vienna). A pre-extraction control virus (phocid alphaherpesvirus) was spiked into each sample according to [45]. The samples were tested for TAHV using a previously described RT-qPCR [30] targeting the viral nucleoprotein open reading frame (Table 1). RusV was tested using the previously described “panRusV-2” RT-qPCR protocol [8] (Table 1).

Samples were tested for LCMV using multiple approaches. First, we used forward and reverse primers from an RT-qPCR recommended by a 2021 European external quality assessment for rodent-borne viruses [46] designed to amplify a region of the S segment (originally published in [47]). We modified these primers based on recently published LCMV sequences from central Europe, and validated the assay using the LCMV-Armstrong reference strain. The primers were LCMV_S1 (5′-GGG ATC CTA GGC TTA TTR GAT-3′) and LCMV_AS1 (5′-GCA CWA TWA TRA CAA TGT TGA T-3′). The reaction was performed as a one-step RT-qPCR with SYBR Green (“SuperScript™ III Platinum™ SYBR™ Green One-Step qRT-PCR Kit”, ThermoFisher Scientific, Vienna, Austria) on a real-time PCR thermocycler, acquiring a melting curve after 40 cycles. Putatively positive samples had an exponential-type amplification curve and amplicon melting temperatures ≥ 80 °C. Putatively positive samples were confirmed with a nested, conventional RT-PCR targeting the S segment, combining primers from two sources [48,49] to amplify a 444 nt product (Table 1).

**Table 1:** Primers and probes used to detect viruses in this study.

Target	Name	Sequence (5′–3′)	Reference
TAHV ^1^	TsF205	CAGGGGAGGTCGTCAATAAT	[30]
	TsR291	AGCACCCATCTAGCCAAATAC	
	TsP256	[FAM]ATAACAACGATCCTTACCATCCACCGGCTA[BHQ1]	
RusV ^1^	RusV_234+	CCCCGTGTTCCTAGGCAC	[8]
	RusV_323-	TCGCCCCATTCWACCCAATT	
	RusV_256_P	[FAM]GTGAGCGACCACCCAGCACTCCA[BHQ1]	
LCMV ^2^	1817V-LCM	ANATGATGCAGTCCATGAGTGCACA	[49]
	2477C-LCM	TCAGGTGAAGGRTGGCCATACAT	
	1902V-LCM	CCAGCCATATTTGTCCCACACTTT	
	2346C-LCM	AGCAGCAGGYCCRCCTCAGGT	
PhHV-1 ^3^	Forward	GGGCGAATCACAGATTGAATC	[45]
	Reverse	GCGGTTCCAAACGTACCAA	
	Probe	[TET]TTTTTATGTGTCCGCCACCATCTGGATC	

^1^ Primers and probes for RT-qPCR reactions to target Tahyna virus (“TAHV”) or rustrela virus (“RusV”). ^2^ A nested conventional RT-PCR using 1817V-LCM and 2477C-LCM as the outer primers in an RT-PCR, and 1902V-LCM and 2346C-LCM as the inner primers in a PCR; used for sequencing. ^3^ Phocid alphaherpesvirus-1 (PhHV-1) was added to samples pre-extraction and used as a nucleic acid extraction control.

### 2.3. Phylogenetic Analysis of LCMV

The amplicon was sequenced by the Sanger method. For phylogenetic analysis, we selected reference sequences from GenBank using the discontinuous megablast search algorithm, and selected sequence matches by eliminating laboratory strains, removing exact duplicates and removing pairs with high sequence identity by an iterative method (e.g., when multiple sequences were obtained from the same hosts at the same location at approximately the same time). The reference strain “Armstrong 53b” (NC_004294) was included in the analysis, and Ryukyu virus (“Rat arenavirus-1” [50], *Mammarenavirus ryukyuense*, NC_039009) was used as an outgroup to root the tree. The phylogenetic tree was inferred over 5000 bootstraps using the TIM3+F+I+I+R2 substitution model, identified with ModelFinder in IQTree2 (v2.2.0.3). The resulting consensus tree was visualized with “ggtree” (v3.10.1) [51] and related packages, including treeio (v1.26.0) and tidytree (v0.4.6), with assistance from the package “ape” (v5.8) in R (v4.3.3).

## 3. Results

The cohort consisted of 393 samples from patients with suspected meningoencephalitis, and 50 samples from patients thought to have tick-borne encephalitis (all negative by RT-PCR and TBEV-reactive IgM). All samples were negative for RusV and TAHV RNA. One sample was RT-qPCR positive for LCMV, collected from a patient living in Vienna in September 2021, with the physician’s indication “suspected encephalitis”.

**Figure 1:**
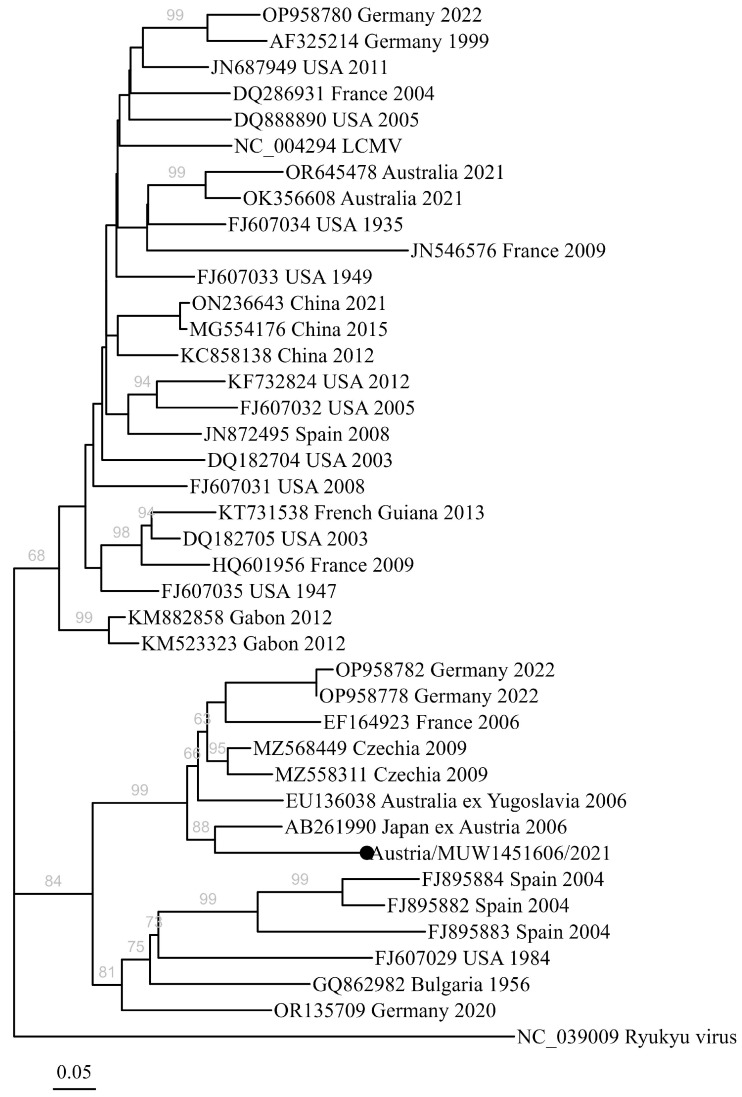
The maximum-likelihood phylogenetic tree of lymphocytic choriomeningitis virus (*Mammarenavirus choriomeningitidis*), including a case of encephalitis in Austria (Austria/MUW1451606/2021, Accession PQ799301, indicated by a black dot). The consensus tree was inferred over 5000 bootstraps, with the TIM3+F+I+I+R2 substitution model identified with ModelFinder in IQTree2 (v2.2.0.3), and nodes with bootstrap support >60% are shown over branches in gray text. Reference sequence names are given as the GenBank accession number, the presumed country of origin (or importation), and the year of collection. The scale bar indicates branch distance in substitutions per site over the 444 nt partial sequence of the viral S segment.

The amplified LCMV sequence (Austria/MUW1451606/2021, GenBank PQ799301) matched only three other sequences using the online megablast algorithm from the NCBI standard database: a sequence from *Mus musculus* in Czechia in 2009 (MZ568449, 85.84% sequence identity); a sequence from *Mus musculus* in Germany in 2022 (OP958780, 83.56% identity); and a sequence from a patient in Spain in 2008 (JN872495, 83.56% identity). A discontinuous megablast further identified the strain from Japan that originated from wild-caught mice in Illmitz, Austria (AB261990) with 85.84% identity [22]; otherwise, the sequence identities to mammarenaviruses in the GenBank (NCBI) database were less than 85%. Phylogenetic analysis placed the sequence in a well-supported clade (97% bootstrap support) with previously described strains from Germany [52], France [53], Czechia [21], and a patient-derived virus sequence from Australia, with the patient having reported travel to “former Yugoslavia” (the so-called “Dandenong strain” [54]). More specifically, the sequence shared a common ancestor (88% bootstrap support) with the Austrian-derived laboratory strain in Japan (Figure 1).

## 4. Discussion

The successful detection of viral genetic material in an infected patient is dependent on the course of disease, the samples tested, and—importantly—the specificity of the test. We identified acute infection with LCMV in the CSF of one patient from a cohort of patients with encephalitis. However, we are cautious when inferring the incidence of LCMV, RusV, and TAHV in Austria based on these observations. Our data suggest that approximately 0.2% of encephalitis patients might have LCMV, and less than 0.2% of patients might have RusV or TAHV. However, each of these viruses is assumed to be seasonally abundant in their enzootic transmission cycles, and therefore spillover to humans would also be seasonal. Furthermore, we must be cautious about associating these viruses with a specific course of disease, and interpreting our data in terms of the sensitivity and specificity of our tests. While biobanks are important resources for retrospective analyses such as ours, they introduce additional considerations in sample processing and virus stability during storage that may reduce the likelihood of detection.

We were screening for evidence of acute-phase virus infection in symptomatic patients. LCMV is known to cause meningitis in patients, causes neurological complications particularly in transplant patients, and is a serious congenital infection [55]. Therefore, it is not surprising that we could link a suspected encephalitis case to an infection with LCMV. However, the situation is not as concrete for TAHV, where the precise description of disease has ranged from simple fever to more complicated neurological symptoms [27,36,37,56]. Our findings support the cumulative evidence that TAHV is a relatively rare infection in humans in Austria [40] and in Europe [27,28,29,41] (particularly in cases with neuropathy), while it has a relatively high seroprevalence in wildlife [34] and is focally and seasonally present in mosquitoes [30,39]. Even less is certain about the course of potential human disease for RusV—it is known to infect and cause encephalitis in many, distantly related mammalian species [9,10], and therefore human infection is suspected, but not yet verified. Our study represents the first extensive screening of human clinical samples for evidence of RusV infection.

Our approach was designed with routine diagnostics in mind, to provide insight into benefits and limitations when implementing routine testing for these viruses among patients with suspected meningoencephalitis. We note that our assembled cohort had been previously screened for a number of viruses in our laboratory, including typical neuroinvasive human viruses, tick-borne encephalitis virus, and other mosquito-borne viruses; however, we have no knowledge about the ultimate diagnosis of these patients, whether, for example, another microbiological laboratory was able to associate bacterial infections as the cause of the disease. Our biobank approach allowed us to perform retrospective analyses with the intention of identifying and characterizing these viruses to improve diagnostics.

The most important caveat for our study is that we relied on PCR-based molecular diagnostic methods. Other molecular approaches for detecting viral nucleic acids nonspecifically, such as “shotgun sequencing” or metagenomics, are becoming more feasible for routine diagnosis [57,58,59]. However, these approaches still have low sensitivity compared to RT-qPCR and require either more sample material, high sequencing depth, and/or a relatively high viral load [57,59]. Importantly, prior to screening, we validated our optimized LCMV RT-qPCR primers to show that they have similar sensitivity compared to the published assay as well as the nested conventional RT-PCR described herein for a laboratory strain of LCMV; and, having identified the virus in a patient sample, we were able to show the similarity in sensitivity of these assays against a “wildtype” strain found in Austria. Additionally, alternative methods, involving serological tests to detect virus-specific IgM or IgG, are not commercially available for our three selected viruses. TAHV has remarkably low sequence diversity over time and across its range in Europe [30], providing confidence that our PCR would have detected the virus. Similarly, we used the assay that has been known to be specific to the clade of RusV identified in Austria in cats [8].

In contrast to TAHV, LCMV is known to have very high sequence diversity [21], complicating diagnostic tests [46]. Very few sequences of LCMV from Central Europe are available [26], and, as mentioned, the single LCMV sequence available linked to Austria had uncertain and indirect provenance [22]. Indeed, all available human- and rodent-derived LCMV sequences must be cautiously interpreted in terms of viral phylogeography, as discussed in [21] (Figure 1). Therefore, designing specific primers for PCR-based testing that will detect local strains remains a major hurdle. To our knowledge, there are few, if any, contemporary isolates of LCMV from Europe available for assay validation. Nonetheless, our sequence fits into a strongly supported clade with viruses from central Europe, described from wild-captured rodents [21,52], specifically sharing a direct common ancestor with the laboratory mice reportedly captured in Austria (Figure 1). We are therefore confident that the sequence represents an endemic strain, although we cannot exclude the possibility that it was travel-associated. However, we believe this sequence will assist in further optimizing diagnostic methods to detect autochthonous cases.

## 5. Conclusions

The rapid expansion of known virus diversity and of newly discovered virus species is evident from the rapidly expanding databases of publicly available virus sequences [60]. Molecular diagnostic tools must keep up with this pace by incorporating new sequence data to update primers and probes, and include tools that are sensitive to novel, emerging, or re-emerging pathogens that may cause similar clinical features in patients—fever, flu-like illness, meningitis, or meningoencephalitis. Our identification of LCMV confirms that human cases occur in Austria, possibly at a low overall incidence, but improved diagnostic methods are necessary to better understand the incidence in the population.

## Data Availability

The original contributions presented in this study are included in the article. Further inquiries can be directed to the corresponding author. The virus sequence generated by the study is available in GenBank, Accession number PQ799301.

## Abbreviations

The following abbreviations are used in this manuscript:

LCMV	Lymphocytic choriomeningitis virus (*Mammarenavirus choriomeningitidis*)
NCBI	U.S. National Center for Biotechnology Information
PhHV-1	Phocid alphaherpesvirus-1
RusV	Rustrela virus (*Rubivirus strelense*)
TAHV	Tahyna virus (*Orthobunyavirus tahynense*)
